# Transcript, protein, metabolite and cellular studies in skin fibroblasts demonstrate variable pathogenic impacts of *NPC1* mutations

**DOI:** 10.1186/s13023-020-01360-5

**Published:** 2020-04-05

**Authors:** Dita Musalkova, Filip Majer, Ladislav Kuchar, Ondrej Luksan, Befekadu Asfaw, Hana Vlaskova, Gabriela Storkanova, Martin Reboun, Helena Poupetova, Helena Jahnova, Helena Hulkova, Jana Ledvinova, Lenka Dvorakova, Jakub Sikora, Milan Jirsa, Marie T. Vanier, Martin Hrebicek

**Affiliations:** 1 Research Unit for Rare Diseases, Department of Pediatrics and Adolescent Medicine, First Faculty of Medicine, Charles University and General University Hospital, Ke Karlovu 2, 120 00 Prague 2, Czech Republic; 2 Laboratory of Experimental Hepatology, Institute of Clinical and Experimental Medicine, Prague, Czech Republic; 3 INSERM U820, Lyon, France; 4 Laboratoire Gillet-Mérieux, Lyon University Hospitals (HCL), Lyon, France

**Keywords:** Niemann-pick type C, Lysosomal storage disease, Proteostasis, Mutant protein, Cholesterol transport

## Abstract

**Background:**

Niemann-Pick type C (NP-C) is a rare neurovisceral genetic disorder caused by mutations in the *NPC1* or the *NPC2* gene. NPC1 is a multipass-transmembrane protein essential for egress of cholesterol from late endosomes/lysosomes. To evaluate impacts of *NPC1* mutations, we examined fibroblast cultures from 26 NP-C1 patients with clinical phenotypes ranging from infantile to adult neurologic onset forms. The cells were tested with multiple assays including *NPC1* mRNA expression levels and allele expression ratios, assessment of *NPC1* promoter haplotypes, NPC1 protein levels, cellular cholesterol staining, localization of the mutant NPC1 proteins to lysosomes, and cholesterol/cholesteryl ester ratios. These results were correlated with phenotypes of the individual patients.

**Results:**

Overall we identified 5 variant promoter haplotypes. Three of them showed reporter activity decreased down to 70% of the control sequence. None of the haplotypes were consistently associated with more severe clinical presentation of NP-C. Levels of transcripts carrying null *NPC1* alleles were profoundly lower than levels of the missense variants. Low levels of the mutant NPC1 protein were identified in most samples. The protein localised to lysosomes in cultures expressing medium to normal NPC1 levels. Fibroblasts from patients with severe infantile phenotypes had higher cholesterol levels and higher cholesterol/cholesteryl ester ratios. On the contrary, cell lines from patients with juvenile and adolescent/adult phenotypes showed values comparable to controls.

**Conclusion:**

No single assay fully correlated with the disease severity. However, low residual levels of NPC1 protein and high cholesterol/cholesteryl ester ratios associated with severe disease. The results suggest not only low *NPC1* expression due to non-sense mediated decay or low mutant protein stability, but also dysfunction of the stable mutant NPC1 as contributors to the intracellular lipid transport defect.

## Background

Niemann-Pick type C (NP-C) disease is an autosomal recessive neurovisceral lysosomal lipid storage disorder with a severe and progressively debilitating course leading to premature death in most patients. The clinical phenotype is highly heterogeneous. First symptoms can be detected at any age from the newborn period to the sixth decade of life. Aside a perinatal, rapidly fatal systemic form, the age at neurological onset is largely predictive of disease severity. Patients are therefore usually categorized into early infantile, late infantile, juvenile and adolescent/adult neurological forms. Most often, progressive neurodegeneration - presenting with ataxia, vertical gaze palsy, gelastic cataplexy, dysarthria, spasticity, psychosis and intellectual decline - is preceded and/or accompanied by hepatosplenomegaly or splenomegaly; prolonged neonatal cholestastic jaundice is another systemic quite common presenting sign [1, 2]. Psychiatric symptoms in adolescent and adult NP-C patients may be prominent and can be easily misdiagnosed as psychosis, schizophrenia, or bipolar disorder [3]. Several adult patients with relatively benign, visceral-only NP-C1, have also been reported [4–6].

The underlying metabolic defect in NP-C is an impaired trafficking of LDL-derived cholesterol from late endosomes/lysosomes (LE/LY). NP-C evolves due to mutations in either *NPC1* [7] or *NPC2* [8] genes. Defects in both proteins result in an identical cellular phenotype that is characterized by an abnormal LE/LY accumulation of unesterified cholesterol (UC) and glycolipids [9]. Mutations in *NPC1* gene are much more common (occurring in about 95% of NP-C patients) [1]. Crystal structures of NPC1 and NPC2 proteins were determined and support the model of the “hydrophobic handoff” of cholesterol from soluble NPC2 to membrane-bound NPC1 [10, 11].

UC trafficking from LE/LY to endoplasmic reticulum depends on both NPC1 and NPC2 [9], but UC egress to mitochondria [12] requires only NPC2. A pathway for UC trafficking to peroxisomes that depends on NPC1 was described [13]. UC departure from LE/LY to mitochondria requires also MLN64/STARD3, a member of STARD family of cholesterol-binding proteins [14]. UC trafficking to mitochondria is not impaired by NPC1 deficiency and, crucially, mitochondrial cholesterol content is increased in NPC1-deficient cells [15] and may contribute to pathogenesis of NP-C disease [16, 17].

The mainstay of NP-C diagnostics is the classic filipin test [18–21] that detects levels of UC in cytoplasm of cultured skin fibroblasts after challenge with LDL, a sensitive but laborious test, combined with mutation analysis. The discovery of oxysterols, lysosphingomyelin isoforms and analogs and bile acid metabolites [22, 23] as biomarkers of NP-C recently enabled screening from peripheral blood.

Efforts to associate biochemical NP-C phenotypes with clinical severity have shown that patients with severe neurological forms of NP-C show a “classic” biochemical phenotype by the filipin and/or cholesteryl esterification tests [24–27] and tend to have low or undetectable levels of immunoreactive NPC1 [27–29]. On the other hand, a number of patients with other clinical phenotypes, more particularly individuals with adult-onset NP-C, have shown a “variant” filipin test [18, 28, 30] and only moderately decreased or normal levels of immunoreactive NPC1 [28, 29]. Biochemical and clinical phenotype associations were determined for several common *NPC1* mutations. For instance, in the homozygous state, the p.I1061T substitution associates with pronounced cellular cholesterol transport abnormalities, a reduced NPC1 level and a juvenile neurological form [19, 27, 31–33]. In contrast, the p.P1007A mutation links to the “variant” biochemical phenotype (close to normal rates of LDL-induced cholesteryl ester formation and variant filipin test) [18, 27, 28, 34], and near normal NPC1 level [28].

The only currently approved drug for NP-C is the glycosphingolipid synthesis inhibitor miglustat (Zavesca, Actelion) [35]. Intrathecal administration of the cholesterol-sequestering drug 2-hydroxypropyl-β-cyclodextrin has achieved impressive long-term effects in a feline NP-C1 model [36, 37]. Clinical trials with this agent using different administration routes are underway, as well as a trial using arimoclomol, an inducer of heatshock proteins 70 and 40 in cells under stress [38].

In an attempt to improve assessment of the disease severity prognosis, we examined the impact of *NPC1* gene variations on its expression, NPC1 protein level and NPC1 subcellular localization, as well as unesterified and esterified cholesterol levels in cultured skin fibroblasts from a cohort of well-characterised NP-C1 patients [3].

## Results

### Clinical phenotypes and NPC1 mutations in the cohort

**Fig. 1:**
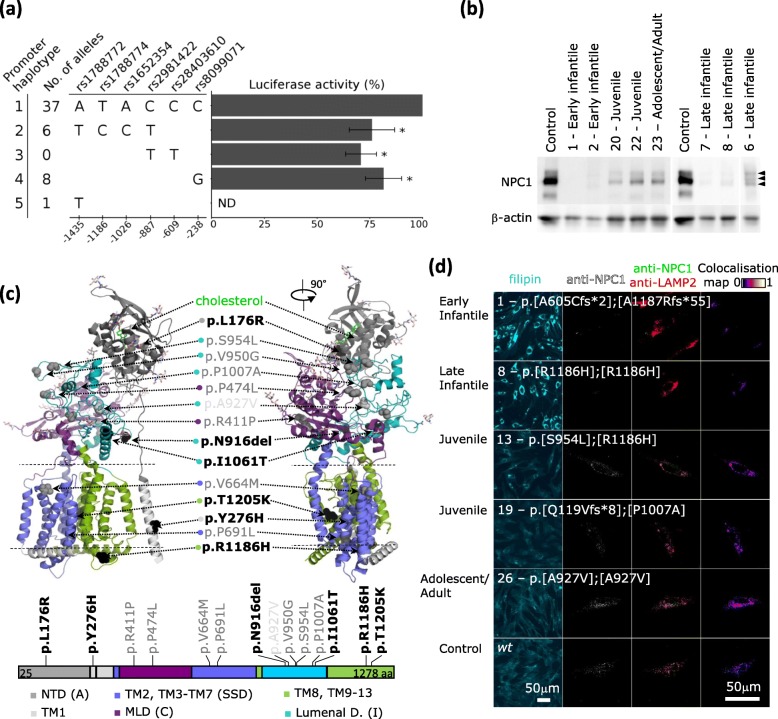
**a***NPC1* promoter haplotype variants and respective luciferase reporter activities in %. Reporter activity of pGL3 basic vector was 0.1 ± 0.1% of Haplotype 1 construct activity. Haplotype 3 is present in controls only. **b** Immunoreactive NPC1 protein in skin fibroblast lines (Western blotting). The cell line numbers and phenotypes are indicated on the top. Equal amount of protein (8 μg) was applied per line. Abnormal banding associated with p.Y276H is indicated by arrowheads. CCD data were used for the quantification. **c** The mutations are depicted using crystal structure of NPC1 protein 3JD8 [40] and the domains are color-coded according to Li [11]. A schematic of primary structure of the mature NPC1 protein is shown below the structure - domain color coding. NTD – N-terminal domain, TM – transmembrane domain, MLD - middle luminal domain, SSD – sterol sensing domain. The most severe mutations are indicated in bold font. Most of the mutations are in lumenal domains I and C (color-coded circles beside the mutation labels). **d** Representative images of human skin fibroblasts of a control and patients with selected forms of the disease. 1st column: direct filipin stained cultures. 2nd column: confocal microscopy images anti-NPC1 signal. 3rd column: merge of anti-LAMP2 (red) and anti-NPC1 (green) signal, 4th column: co-localization overlay maps. Values 0 - 1 of the pixels are displayed using lookup table LUT 0–1. All images were processed equally

Genotypes and phenotypes of the 26 patients are summarized in Table 1 and Table S1. Detailed data on clinical phenotypes are available in [3] for 21 of them (for correspondence see Table S1). The phenotype classification of patients #5 and #24 was revised and reclassified according to Nadjar and colleagues [39] as late infantile and adolescent/adult, respectively. Juvenile NP-C was by far the most common clinical phenotype. Our cohort also comprised 4 patients with the early infantile form, 5 patients with the late infantile form and 4 patients with the adolescent/adult form. The most frequent *NPC1* mutations in our cohort were p.R1186H (*n* = 12, 2 homozygotes), p.S954L (*n* = 10), and p.A927V (*n* = 6, 3 homozygotes). All three are common in European populations. The two variants usually reported as the most prevalent in populations from the Western world, p.I1061T (*n* = 2) and p.P1007A (*n* = 4), were present in compound heterozygosity with other mutations. One patient was a compound heterozygote for two frameshift mutations. The effects of missense mutations p.S954L and p.P1007A were deduced from samples of 7 patients, who carried presumably null frameshift mutations on the other allele. Similarly, impacts of less severe mutations were estimated on the background of known and well characterised severe mutations. The positions of the mutations are highlighted in the NPC1 structure in Fig. 1c.

**Table 1:** Cohort of NP-C1 patients stratified by NP-C clinical phenotypes and corresponding genotypes and results of the cell analyses

Patient No.	NP-C Clinical Phenotype	Genotype Deduced NPC1 protein change (ref. NP_000262.2)	NPC1 Promoter haplotype	Allele expression ratio (%)	NPC1 mRNA level (qPCR)	Relative NPC1 protein amount by Western blot	Colocalization object Pearson’s coefficient	UC/CE ratio	Direct Filipin fluorescence vs. control	LDL-induced Cholesterol esterification rate	Evaluation of vesicular cholesterol by diagnostic filipin test	“NP-C Biochemical Profile”
1	Early infantile	p.[A605Cfs*2];[A1187Rfs*55]	1/2	**10**/**90**	0.29 ± 0.15	0.00 ± 0.00	0.07	5.8 ± 0.6	3.0 ± 0.8	< 10	strong	CLA
2	Early infantile	p.[N916del];[P1245Rfs*12]	1/1	50/**50**	0.56 ± 0.04	0.05 ± 0.00	0.17	8.1 ± 1.7	2.6 ± 1.0	< 10	massive	CLA
3	Early infantile	p.[I1061T]; [T1176_S1196del;S1197_V1198ins15]^$^	1/1	63/37	1.26 ± 0.81	0.09 ± 0.01	0.12	7.0 ± 0.6	2.3 ± 0.9	–	–	–
4	Early infantile	p.[R1186H];[T1205K]	1/4	54/46	0.49 ± 0.21	0.04 ± 0.00	0.07	15.5 ± 1.0	2.7 ± 0.5	< 10	massive	CLA
5 Sb	Late infantile	p.[Y276H];[R1186H]	1/1	48/52	0.87 ± 0.42	0.08 ± 0.01	0.28	10.2 ± 1.5	1.7 ± 0.2	< 10	massive	CLA
6 Sb	Late infantile	p.[Y276H];[R1186H]	1/1	49/51	0.66 ± 0.45	0.14 ± 0.03	0.24	7.7 ± 0.8	1.5 ± 0.5	< 10	massive	CLA
7	Late infantile	p.[R1186H];[R1186H]	1/4	–	0.51 ± 0.31	0.03 ± 0.00	0.15	11.9 ± 1.8	3.0 ± 0.8	–	–	–
8	Late infantile	p.[R1186H];[R1186H]	1/1	–	1.40 ± 0.42	0.05 ± 0.01	0.27	7.1 ± 1.3	2.1 ± 0.8	–	–	–
9	Late infantile	p.[P1007A];[R1186H]	1/1	49/51	0.92 ± 0.30	0.85 ± 0.08	0.72	3.2 ± 0.7	1.3 ± 0.4	300	important	INT
10	Juvenile	p.[S954L];[R1186H]	1/1	51/49	0.88 ± 0.14	0.18 ± 0.01	0.42	3.3 ± 0.6	1.6 ± 0.3	220	strong	CLA
11	Juvenile	p.[S954L];[R1186H]	1/1	55/45	1.60 ± 0.63	0.28 ± 0.01	0.62	3.7 ± 0.9	0.9 ± 0.3	120	massive	CLA
12 Cs	Juvenile	p.[S954L];[R1186H]	1/1	51/49	1.44 ± 0.19	0.29 ± 0.05	0.63	2.1 ± 1.0	1.3 ± 0.2	–	–	–
13 Cs	Juvenile	p.[S954L];[R1186H]	1/1	52/48	0.80 ± 0.27	0.36 ± 0.04	0.62	4.3 ± 0.8	1.2 ± 0.3	185	abnormal	INT
14	Juvenile	p.[P474L];[P691L]	1/2	48/52	0.93 ± 0.57	0.33 ± 0.03	0.77	2.7 ± 0.8	0.9 ± 0.4	25	massive	CLA
15	Juvenile	p.[V950G];[P1007A]	1/4	71/29	0.86 ± 0.49	0.32 ± 0.00	0.77	1.5 ± 0.1	0.8 ± 0.2	510	very significant	INT/VAR
16	Juvenile	p.[R411P];[P1007A]	1/4	51/49	1.29 ± 0.75	0.44 ± 0.01	0.79	3.4 ± 0.2	0.9 ± 0.1	1195	moderate but significant	VAR
17	Juvenile	p.[R411P];[S954L]	1/4	51/49	0.52 ± 0.18	0.52 ± 0.06	0.68	4.6 ± 0.9	1.3 ± 0.2	–	–	–
18	Juvenile	p.[L176R];[S954L]	1/1	48/52	0.60 ± 0.32	0.72 ± 0.08	0.75	3.5 ± 0.6	1.5 ± 0.3	320	strong	INT
19	Juvenile	p.[Q119Vfs*8];[P1007A]	1/1	**25**/75	1.60 ± 0.67	0.74 ± 0.03	0.67	3.5 ± 0.6	0.6 ± 0.1	580	moderate but significant	VAR
20	Juvenile	p.[V270Sfs*40];[S954L]	1/1	**36**/64	0.66 ± 0.25	0.17 ± 0.03	0.58	4.2 ± 0.9	1.2 ± 0.2	–	–	–
21	Juvenile	p.[E575Rfs*17];[S954L]	1/5	**11**/89	0.30 ± 0.13	0.25 ± 0.02	0.67	3.1 ± 0.2	1.1 ± 0.3	220	massive	CLA
22	Juvenile	p.[A605Cfs*2];[S954L]	1/2	**11**/89	0.30 ± 0.14	0.29 ± 0.03	0.71	3.9 ± 0.1	1.1 ± 0.2	240	marked	INT
23	Adolescent/Adult	p.[Q905Rfs*31];[S954L]	1/1	**10**/90	0.25 ± 0.09	0.30 ± 0.03	0.66	2.7 ± 0.5	0.9 ± 0.1	–	–	–
24 Sb	Adolescent/Adult	p.[A927V];[A927V]	2/4	52/48	0.90 ± 0.37	0.49 ± 0.11	0.69	1.4 ± 0.1	1.1 ± 0.4	834	moderate but significant	VAR
25 Sb	Adolescent/Adult	p.[A927V];[A927V]	2/4	–	0.48 ± 0.30	0.73 ± 0.00	0.69	2.1 ± 0.2	1.2 ± 0.3	337	moderate	VAR
26 Sb	Adolescent/Adult	p.[A927V];[A927V]	2/4	50/50	1.11 ± 0.59	0.74 ± 0.01	0.67	3.3 ± 0.1	1.0 ± 0.3	870	moderate but significant	VAR
Control levels (*n* = 3)		wt		50/50	0.95 (0.35–1.34)	0.94 (0.56–1.27)	0.77 (0.71–0.80)	3.2 (2.3–3.7)	1.00 (0.80–1.33)	2950 ± 1200	*absent/very low*	normal

Values in “allele expression ratio” are in the order corresponding to the order of alleles as in the column “deduced protein change”. Bold font indicates frameshift mutations. ^$^ effect of the splice mutation on protein assumed from cDNA analysis in Ribeiro et al. [28] LDL-induced cholesterol esterification rates are expressed in pmol cholesteryl-[^3^H]oleate formed/mg protein/4.5 h. A column linking the cell lines to patients of the cohort of reference [3] is available in supplementary Table S1. Abbreviations: *CLA* classical, *INT* intermediate, *VAR* variant [18], *UC* unesterified cholesterol, *CE* cholesteryl esters, *Sb* sibling, *Cs* cousin

### Analysis of the NPC1 promoter region

We identified 6 common variants clustered into 5 haplotypes (Fig. 1a) in the *NPC1* promoter region in the cell lines of patients and controls. Haplotypes 2, 3, and 4 contained 4, 2, and 1 sequence variants, respectively, that were not present in the reference sequence (haplotype 1). Promoter fragments corresponding to haplotypes 2, 3, and 4 had 20–30% lower luciferase reporter activities than haplotype 1. The differences were statistically significant (p = < 0.001). Rare haplotype 5 (one allele in the cohort only) was not tested by the reporter assay.

### Expression of the NPC1 transcripts

*NPC1* relative expression measured by two TaqMan assays (Hs00264835_m1 and Hs00975249_m1) were highly correlated (correlation coefficient r = 0.92, *p* < 0.0001), therefore, only results of Hs00264835_m1 assay were used for analysis. Relative expression of *NPC1* in patients was comparable to controls. Lowest levels of *NPC1* expression (0.25-0.30) were found in samples carrying a frameshift mutation (#1, #21, #22, #23).

*NPC1* allelic expression ratios in fibroblasts carrying two missense mutation were ~ 50/50 (Table 1) with the exception of 70/30 ratio in patient #15. This discrepancy was likely caused by combination of promoter haplotypes 1 and 4 and/or altered transcript stability. In patients carrying a nonsense and a missense mutation, expression ratios were usually skewed in favour of the missense mutation (90/10–65/35). In patient #1, who carried two frameshift mutations p.[A605Cfs*2];[A1187Rfs*55], the allelic ratio was shifted in favour of the latter (presumably more stable) allele (10/90).

We did not identify any major promoter haplotype-linked differences in allelic *NPC1* expression. For example, there was identical expression of both *NPC1* alleles in two siblings #24 and #26 homozygous for p.A927V and heterozygous for promoter haplotypes 2 and 4 and SNP rs1140458 in the coding region.

### Semi-quantitative measurement of NPC1 protein in skin fibroblast lines

**Fig. 2:**
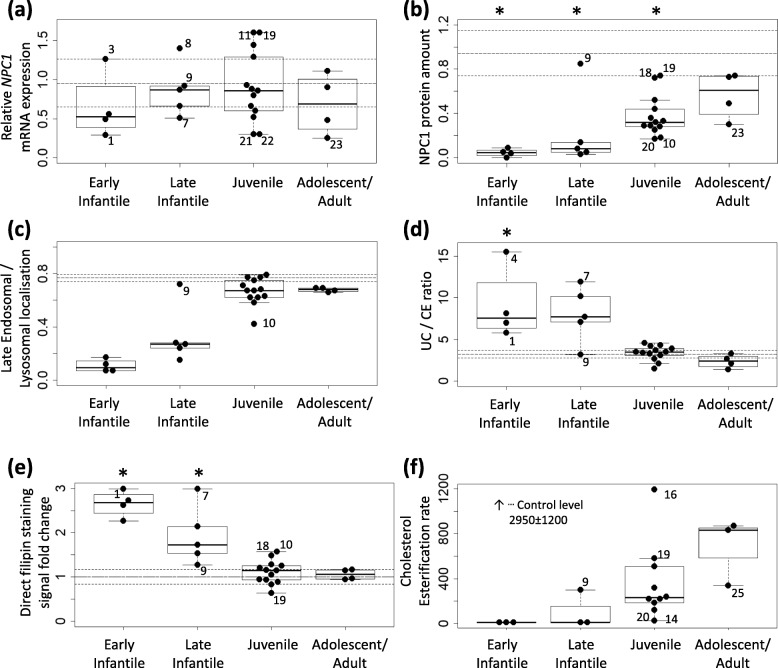
Overview of NP-C1 patient fibroblasts analyses. Strip plots show results of assays for the four phenotypic subgroups. Solid circles represent individual patient cell lines. Numbers adjacent to the circles represent patient number. The long-dashed and dashed horizontal lines represent the average control levels and standard error of means, respectively. Asterisks above the boxplots (2a, 2b, 2d, 2e) mark groups that differ significantly from controls. The groups were compared by one-way ANOVA test followed by post-hoc Tukey’s HSD test adjusted for unequal sample sizes. **a***NPC1* mRNA expression level, **b** NPC1 protein level by Western Blotting, **c** endosomal / lysosomal localisation of NPC1 protein. NPC1 vs. LAMP2 Object Pearson’s colocalization coefficient, **d** unesterified cholesterol / cholesterol ester ratio by MS/MS, **e** direct filipin staining of cellular unesterified cholesterol, **f** cholesterol esterification rate of LDL-derived cholesterol

Fibroblasts with the lowest levels of the NPC1 protein were from patients affected by the early or late infantile forms of NP-C. On the contrary, patients with the highest levels of NPC1 protein presented with the adolescent/adult disease (Table 1, Fig. 1b, Fig. 2b and Figure S1). Patient #9 (p.[P1007A];[R1186H]) with relatively high amount of NPC1 protein and a late infantile phenotype was an exception. The lowest amount of NPC1 protein was found in samples from patients #1 (p.[A605Cfs*2];[A1187Rfs*55]), #2 (p.[N916del];[P1245Rfs*12]), #7 and #8 (p.[R1186H];[R1186H] homozygotes). These findings correspond well with the severe impact of the latter mutation. Samples of the two siblings #5 and #6 (p.[Y276H];[R1186H]) repeatedly displayed slightly altered banding patterns on Western blots compared to controls (Fig. 1b).

### Confocal microscopic co-localization studies

Subcellular localization of the mutant NPC1 was difficult to assess in cells with extremely low expression of the protein (e.g. fibroblasts carrying p.R1186H or frameshift mutations). In these cells the NPC1 fluorescent signal was indistinguishable from the background (Table 1 and Fig. 1d). For this reason we did not evaluate possible co-localization of NPC1 with the ER-marker protein disulfide isomerase (PDI) that was tested by others [41]. Localization of NPC1 was evaluated only in cell lines expressing higher amounts of the mutant protein. We presume that in cell lines carrying heterozygous frameshift *NPC1* mutations the detected protein is expressed from the other allele (e.g. p.S954L and p.P1007A). The signal from p.A927V, p.S954L and p.P1007A NPC1 mutant proteins colocalized with the late endosomal/lysosomal (LE/LY) marker LAMP2 (Table 1, Fig. 1d and Figure S1).

### Concentrations of UC and CE, direct quantitative filipin staining, diagnostic filipin test and LDL-induced rates of cholesteryl ester formation in native cultured skin fibroblast lines

We evaluated UC/CE ratio instead of their separate values (Table S1) as we expected increase of UC and decrease of CE due to the impaired metabolic turnover of cholesterol by the NPC protein mutation. UC/CE ratios were elevated (Table 1, Fig. 2d) in 8 patients with the most severe phenotypes (early infantile, late infantile), while in patients with milder phenotypes the values overlapped with controls. However, in patient #9 with late infantile NP-C (p.[P1007A];[R1186H]) UC/CE ratios were comparable to controls.

The strongest filipin signal under steady-state conditions was detected in cells homozygous or compound heterozygous for null and severe *NPC1* mutations, such as p.R1186H or p.T1205K (Table 1 and Fig. 1d). Conversely, a low direct filipin staining signal was observed in cells compound heterozygous for a null allele and p.S954L, also showing a good level of NPC1 protein (patients #19–23). Of note, in cells from compound heterozygotes for p.P1007A, signs of perinuclear cholesterol accumulation by filipin staining were only revealed after preincubation of the cell cultures with lipoprotein-deficient serum followed by LDL loading (diagnostic filipin test).

LDL-induced early rates of cholesteryl ester formation were often close to nil and < 150 pmol/4.5 h/mg protein (classical profile) in all cell lines with very low NPC1 protein levels, and showed higher values with a wide variation in the other cell lines, falling into two previously described categories [20], intermediate (values 10–15% of normal) and variant (> 15% of normal, i.e. > 500 pmol/4.5 h/mg protein) (Table 1).

## Discussion

In an attempt to correlate molecular and biochemical phenotypes with the severity of the neurological disease in NP-C1 disease, we applied to fibroblast cell lines from well characterized NP-C1 patients [2] a more extensive array of molecular and biochemical tests than previously reported.

First we searched for *NPC1* promoter variants that could influence transcription efficiency. *NPC1* transcription is largely constitutive [42, 43] and its promoter contains a putative TFEB element [44]. Its expression is also modulated by sterol regulatory element–binding protein (SREBP) pathway via SREBP-binding elements in the *NPC1* promoter [41, 45]. In steroidogenic cells *NPC1* expression is also regulated by the 3′,5′-cyclic adenosine monophosphate pathway [46]. None of the variants defining the four haplotypes identified in our cohort were located in the putative regulatory elements in the promoter [41, 44]. Three haplotypes led to mild to moderate decreases of luciferase reporter activity (down to 70% of the wild-type activity in haplotype 1 (Fig. 1a)). The effects of the haplotypes in vivo could be evaluated only indirectly from their associations with skewed allelic expression, transcript levels and clinical phenotypes. Neither of the haplotypes could be convincingly associated with more severe phenotype or with skewing of allelic expression ratios in patients carrying missense mutations. This led us to conclude that in our cohort causative mutations in the coding region were the major determinant of the phenotype and promoter haplotypes played a minor role, if any.

Allelic expression ratios in compound heterozygotes for two missense *NPC1* mutations suggested comparable stability of the missense transcripts. On the other hand, ratios in patients carrying a null and a missense mutation were usually skewed in favour of the missense transcript, probably due to the degradation of the nonsense transcript by nonsense-mediated decay (NMD) [47]. This result is supported by our concomitant findings of decreased *NPC1* mRNA level in the presence of a nonsense mutation. It would also explain that patients carrying certain “mild” *NPC1* mutations even in compound heterozygosity with a null mutation, may exhibit a late, adolescent/adult onset neurological phenotype [23, 39], as seen in patient #23.

Additionally, we evaluated the relationship between the clinical phenotype (Fig. 2, Figure S2) and the cellular UC/CE ratios and quantitative filipin staining under steady-state conditions. The differences between controls and early infantile forms have been judged to be statistically significant for both items, and with late infantile forms for filipin signal. But there was an overlap between values observed in controls and in patients with juvenile and adolescent/adult-onset NP-C1 phenotypes. Such an observation was certainly expected for cell lines categorized with a “variant” biochemical profile (and possibly even for those with an “intermediate” profile) from diagnostic filipin testing and early rate of LDL-induced cholesteryl ester formation [18, 20, 30]. In the latter tests, alterations in cellular cholesterol trafficking are strongly amplified by initial upregulation of LDL-receptors followed by massive LDL loading. This amplification is required for diagnostic purposes, and it also illustrates the existence of an NPC1 deficit, but it does not necessarily reflects steady-state conditions. Particularly, it has been well shown that kinetics of LDL-induced cholesteryl ester formation show an initiation delay, but not a block [20, 48].

Levels and subcellular localization of mutant NPC1 proteins constitute further important pieces of information, for which limited data are yet available. A recent publication [49] evaluated the impact of NPC1 missense mutations on NPC1 protein trafficking along the secretory pathway, however, these analyses were performed in COS-1 cells overexpressing NPC1 constructs, not native mutant fibroblast cell lines. In our study, similar to other published cohorts [28, 32], fibroblast protein levels inversely correlated with the severity of the patients’ neurological phenotype. For missense mutations, this observation likely reflects the degradation of misfolded mutant NPC1 proteins [50]. The p.R1186H in homozygous state leads to a severe reduction of NPC1 protein (this study and [32]). It has constantly been associated with a severe (early infantile or late infantile) neurological phenotype, [32, 51] as well as pronounced abnormalities of cellular cholesterol processing [32, 51]. In compound heterozygosity, the observed protein level appears to depend on the second allele. It was low in combination with p.T1205K and p.Y276H alleles, both corresponding to a severe clinical phenotype. In the p.[R1186H];[Y276H] cell line, the observed NPC1 protein must originate from the allele carrying p.Y276H. The cause of the abnormal banding is not obvious; it is probably not caused by abnormal glycosylation as there is no N-glycosylation site in the vicinity of p.Y276H mutation. Protein levels were intermediate for p.R1186H in combination with p.S954L in 4 patients with a juvenile clinical phenotype. There was, however, a notable lack of correlation in line #9 (p.[R1186H];[P1007A]) between moderately high levels of NPC1 protein properly localising to LE/LY compartment and the late infantile phenotype of the patient suggesting that p.P1007A alleles express a relatively stable NPC1 protein. In the two cell lines (#9 and #19) with a combination of a p.P1007A allele and a severe allele, not only protein levels but also direct filipin staining and UC/CE ratios were at the control level implying that the cholesterol transport function is less compromised. The latter findings are in good agreement with the well-known observation of a correlation between p.P1007A and a near normal cholesterol trafficking (so-called “variant”) profile, usually even in a compound heterozygous status [18, 27, 30]. From a clinical point of view, the few patients homozygous for p.P1007A have shown a juvenile or adult neurological phenotype [19, 28, 39], but also other more severe phenotypes when associated with another allele [3, 19, 28, 30, 33]. In cell line #3 with p.[I1061T];[T1176_S1196del;S1197_V1198ins15], the above-average mRNA levels and very low NPC1 protein levels (80–90% reduction) can be attributed to p.I1061T, as such levels are typical for p.I1061T homozygotes [28, 31]. Cells carrying p.S954L and a null mutation, corresponding to 4 patients with a juvenile or adolescent/adult disease form, showed moderate levels of NPC1 protein, with very decreased transcript levels for all four null alleles. In all four lines, the NPC1 signal strongly co-localized with the lysosomal marker (LAMP2) suggesting that mutant p.S954L protein reaches the LE/LY compartment. Similarly, in 3 homozygotes for mutation p.A927V with late onset NP-C, the mutant protein localized correctly to LAMP2 positive vesicles. This mutant was previously characterized as showing a variant filipin pattern [3]. The later onset clinical phenotypes observed for all patients carrying p.S954L and p.A927V strongly suggest that these mutant proteins indeed have a residual functionality.

## Conclusions

In summary, there is no single cellular biomarker that can reliably predict NP-C1 severity. As shown here, testing of multiple laboratory variables provides a granular view of the cellular impacts of *NPC1* mutations and can improve severity predictions. Overall, low immunoreactive NPC1 protein levels were the best predictor of the severity of the disease. Our results are in agreement with the general assessment that the amount and residual cholesterol transport capacity of mutant NPC1 are major predictors of clinical severity; however, a number of patients with an adult-onset neurological disease whose fibroblasts display a severe block of lysosomal cholesterol egress have been described (e.g. some homozygotes for p.I1061T, patients #15, 17, 23, 24 in the Nadjar et al. study, [39] and patient 2 in Table 5 in [3]), that would require further investigations. Mutant NPC1 proteins, particularly in patients with later-onset phenotypes, retain residual functional capacity and thus represent attractive targets to therapeutic proteostatic modulation [32, 52, 53]. The results thus indicate a potential of the combinatorial approach for evaluation of impacts of NPC1 mutations.

## Methods

### Subjects

We studied 26 skin fibroblast cell lines of NPC1-deficient patients from 23 non-related families. Twenty-one of the patients were described by Jahnova and co-workers, [3] to which we refer the reader for patients’ detailed clinical and laboratory characteristics. These cell lines are highlighted in Table S1. Cell lines from 8 healthy controls were also used.

### Analysis of promoter sequence and determination of haplotypes

A 1.7 kb *NPC1* promoter fragment was amplified from genomic DNA of all NP-C patients in the cohort and Sanger sequenced on automated capillary sequencers (ABI Prism 3100-Avant or 3500xL Genetic Analyzer; Life Technologies). PCR products containing sequence variants were cloned using a TA cloning kit (TOPO-TA, Thermo Fisher Scientific) and individual clones were sequenced. Haplotypes were determined from variants found in individual clones. Sequence of primers used for amplifications and sequencing are available upon request.

### Reporter gene assays – promoter activity

Reporter plasmid constructs were prepared as follows. PCR products containing a 1688 fragment of the predicted *NPC1* promoter were amplified using primers with overhangs carrying *Xho*I sites. Primer sequences were derived from the genomic sequence of chromosome 18 (GenBank accession No. NG_012795.1).

Amplified promoter fragments were inserted in both sense and antisense orientations into the *Xho*I site of pGL3basic (luc2CP/Hygro) vector (Promega), at the polylinker site upstream from the firefly luciferase reporter gene, generating pGL3-NPC1. Four constructs carrying sequence variants forming the four *NPC1* haplotypes in the same manner were created, generating pGL3-NPC1-Hap1 to Hap4.

HepG2 (human hepatoblastoma) cells were grown in Opti-MEM (Agilent Technologies) medium supplemented with 10% (v/v) foetal bovine serum (FBS) in 25 cm^2^ flasks at 37 °C, and 5% CO_2_. A total of 5 × 10^4^ HepG2 cells were seeded per well into 24-well culture plates 24 h prior to transfection. HepG2 cells were transfected with 166 ng per well of each construct or the empty pGL3 using the Tfx^TM^ – 20 and FuGene HD Transfection Reagent (Promega). pRL-TK vector (Promega) harboring the Renilla luciferase gene was cotransfected as an internal control to normalize transfection efficiency.

Experiments were done in triplicate and each transfection was repeated independently at least three times. After 48 h, transfected cells were washed with PBS and lysed with 100 μl of Passive lysis buffer (Promega). Luciferase reporter activity was assayed using a Dual-Luciferase Reporter Assay System (Promega). The intensity of chemiluminescence was measured in the supernatant using a luminometer (Berthold Technologies). The results were analyzed using one-tailed *t*-test.

### Quantitative RT/PCR

Total RNA was isolated from skin fibroblast cultures using standard procedures [54] and reverse transcribed using High Capacity cDNA Reverse Transcription Kit (Life Technologies).

Two TaqMan Gene Expression Assays (Applied Biosystems), Hs00264835_m1 and Hs00975249_m1, were used for relative qPCR measurements of *NPC1* transcript abundance. To identify suitable endogenous controls cDNA samples from 7 NPC1-deficient patients and 7 control individuals using Human Endogenous Control Array (Applied Biosystems) were tested. The readouts were analyzed using NormFinder (Aarhus University Hospital, Aarhus, Denmark) and beta-2 microglobulin *B2M* gene was selected as an endogenous control. *NPC1* relative expression was measured using 2^-∆∆Ct^ method [55]. The results were expressed relative to *NPC1* expression of a control reference sample which was assayed simultaneously with each batch of patient samples. All analyses were carried out using Applied Biosystems 7900 Real Time PCR system (Applied Biosystems).

### Determination of allele expression ratios by deep sequencing

*NPC1* transcript fragments containing pathogenic or non-pathogenic variants were amplified from the patient cDNAs by PCR as described previously [3]. The minimum length of PCR products was 300 bp. PCR libraries were prepared using Nextera XT DNA Sample Preparation Kit (Illumina) and indexed using Nextera XT Index Kit (Illumina). The libraries were sequenced using MiSeq Reagent Nano Kit v2 (2*250) and Nano Flow Cell 500 cycles on MiSeq Sequencer (Illumina).

The reads were aligned to the *NPC1* reference sequence (NM_000271) and analyzed using NextGENe software package (SoftGenetics). Only samples with depth greater than 800 reads were included in the analysis. The ratio of reads containing wild-type and variant sequences was calculated after removal of reads carrying probable sequencing errors at the site of evaluated variant. Heterozygosity for SNP c.2793C > T rs1140458 was used for the determination of allele ratio in samples #24 and #26 (genotype p.[A927V];[A927V]). Both the variant and the mutation localize to *NPC1* exon 18.

### Cell culture

Fibroblasts from patients and control subjects were cultured according to routine procedures in DMEM/10% FBS and 5 g/l glucose and penicillin/streptomycin antibiotics in 25 cm^2^ culture flasks and maintained in the same medium for all experiments except for diagnostic filipin testing and LDL-induced cholesterol esterification assays. For non-microscopic studies, confluent cells were PBS washed and harvested by scraping into PBS and centrifuged. The cell pellet was kept frozen at − 20 °C until use.

### Western blot analyses

The samples were sonicated and the protein content was determined by Direct Detect spectrometer (Merck Millipore). Samples were mixed with 6X SDS non-reducing sample buffer (0.35 M Tris, pH 6.8, 10% SDS, 30% glycerol, 0.012% bromophenol blue) and non-boiled samples (8 μg of whole cell lysates) were resolved by 10% SDS-PAGE electrophoresis under non-reducing conditions. Protein samples were transferred onto Immobilon-P PVDF membrane (Merck Millipore) using a semi-dry electroblotter (Biotec-Fischer). Reversible Ponceau S was applied to check equal loading of gels. Immunodetection of NPC1 and beta-actin proteins was performed using a rabbit monoclonal anti-NPC1 antibody (ab134113, Abcam) at a 1:3000 dilution, and a mouse monoclonal anti-beta-actin antibody (mAbcam 8226, Abcam) at a 1:4000 dilution, respectively. Detection was performed by chemiluminiscence using SuperSignal West Femto Maximum Sensitivity Substrate (Thermo Scientific). Image capture was carried out using ChemiGeniusQ analysis system and GeneSnap software (Syngene, Cambridge, UK). Images were analysed using GeneTools software package (Syngene).

### Confocal microscopy

For confocal microscopic co-localization studies, the cells were seeded onto BD Falcon Cultures Slides (Becton Dickinson). Next day the fibroblasts were washed, fixed with ice-cold methanol, blocked with 5% FBS in PBS and co-labelled rabbit monoclonal anti-NPC1 (1:100, ab134113, Abcam) and mouse monoclonal anti-LAMP2 (1:500, H4B4, Iowa Hybridoma Bank) antibodies at 4 °C overnight. Secondary antibodies were donkey anti-IgG anti-mouse alexafluor555 or anti-rabbit alexafluor488 conjugates (Pierce) diluted 1:1000. Leica SP8X laser scanning confocal system equipped with 470 nm–670 nm 80 MHz pulse continuum White Light Laser 2 and HC PL APO 63x/1.40 OIL CS2 (W.D. 0.14 mm) objective was used to image the samples. Image acquisition conditions were: excitation at 496 nm or 553 nm, one voxel 42.2 × 42.2 × 130 nm, 7 Z-steps (fulfilling Nyquist sampling theorem), Hybrid detectors at 503–553 nm or 566–650 nm. Acquired confocal images were deconvolved using theoretical point spread function in Huygens Professional software (Scientific Volume Imaging - SVI, Hilversum, The Netherlands). Overlay colocalization maps and Object Pearson’s coefficients were computed using Huscript (SVI), the grayscale maps were converted to colour coding look-up table (LUT) in Fiji/ImageJ software (NIH, Bethesda).

### Direct quantitative filipin staining

Cells were seeded onto BD Falcon culture slides and cellular cholesterol accumulation was visualized and quantified after direct filipin staining [56]. Briefly, cells were cultured under steady-state conditions as stated above, washed with PBS, fixed using 4% paraformaldehyde and stained with 0.1 mg/ml filipin (Sigma) in PBS, prepared by dilution of a filipin DMSO stock solution prepared the same day. To decrease filipin photobleaching ProLong Gold Antifade Mountant (LifeTech) was used as antifade mountant. Slides were examined using a Nikon Eclipse TI fluorescence microscope equipped with DAPI filter set and all photographed at constant 100 ms exposure time. The exposure time was optimized using the most intensive cell samples to prevent pixel saturation. The fluorescent signal density of individual cells (*N* = 10 per cell line) was manually acquired using ImageJ (NIH, Bethesda). Average corrected total cell fluorescence per one cell line was calculated. The presented values reflect fold change relative to average of controls.

### Diagnostic filipin testing

Diagnostic filipin testing was performed at the time of individual diagnostic process as described in Vanier et al. [20] A main difference with the direct steady-state procedure was that dual culture slides (Lab-Tek chambers) with cells from each fibroblast line were first incubated in medium supplemented with 10% lipoprotein-deficient serum (LPDS) for 3 days, and then challenged for 24 h with (1) medium supplemented with LPDS and 50 μg/ml purified human LDL and (2) medium supplemented with 10% fresh total human serum, prior to filipin staining. A control and a typical NP-C cell lines were included in each diagnostic experiment. Fluorescence microscopic examination was performed using a selective DAPI filter and expert visual evaluation done as discussed by Vanier and Latour [18].

### LDL-induced early rates of cholesteryl ester formation

The test, including complex cell culture conditions, was performed exactly as described by Vanier et al. [20] Esterification rates in Table 1 show values of cholesteryl-[^3^H] oleate formed/4.5 h/mg protein. Classification of the cell lines into biochemical classical, intermediate or variant NP-C profiles [18, 20] was based on combined evaluation of diagnostic filipin testing and results of this assay (both performed in MTV’s laboratory).

### Mass spectrometry

Unesterified cholesterol and cholesteryl ester levels were analysed in fibroblast cultures cultured under steady-state conditions. Briefly, cells were washed with PBS and harvested using trypsinization and centrifugation. The harvested cells were homogenized in water by sonication and extracted in chloroform:methanol (2:1, v/v) mixture [57]. The extract was filtered via hydrophilic PTFE filters (Millex LH filters, Millipore), dried under the stream of nitrogen and processed for mass spectrometry analysis by modified method of Liebisch et al. [58] Major changes were in replacement of chloroform by hexane and acetylchloride with propionylchloride in the derivatization mixture and the rest of the procedure remained unchanged. Mass spectrometry analysis was performed on triple quadrupole tandem mass spectrometer AB/MDS SCIEX API4000 with electrospray ionization coupled with Agilent 1290 Infinity UPLC. 200 pmol of d7-cholesterol and 40 pmol of C17:0 cholesterol ester were used as internal standards for UC and CE quantitative analysis.

### Data analysis and statistics

Statistical computing were performed in R software v3.5.1 or STATISTICA v12. *P*-values < 0.05 were considered statistically significant.

## Supplementary information

**Additional file 1: Table S1.** Cohort of NP-C1 patients. Correspondence with patients in Jahnova et al. [3] and full genotypes. **Supplementary Figure 1**: Scatter plot of NPC1 protein level semi-quantified byWestern blot analysis vs. object Pearson colocalization coefficient of NPC1 and LAMP2. Data labels indicate promoter haplotype allele combinations and deduced effects of the mutations in NPC1 protein. The dashed lines mark levels in controls (AVG ± SEM). **Supplementary Figure 2**: Mutant NPC1 residual function analyses. A scatter plot of Free Cholesterol / Cholesterol Ester ratio vs. direct filipin staining signal fold-change reflecting impaired cholesterol transport caused by NPC1 mutations. The combination of these markers suggest separation of severe infantile phenotypes. Solid circles represent individual patient cell lines and their grey level indicate clinical phenotype. Numbers in bold adjacent to the circles represent values of the LDL-cholesterol esterification rate assessed in the lab. of Dr. Vanier in Lyon. The dashed lines mark levels in controls (AVG ± SEM).

## Data Availability

The dataset is summarized in the figures and tables. The raw data used and/or analyzed during the study are available from the corresponding author on reasonable request.

## Abbreviations

UC/CE: Unesterified cholesterol/cholesterylester
LE/LY: Late endosomal/lysosomal
LDL: Low-density lipoprotein
LPDS: Lipoprotein-deficient serum
FBS: Foetal bovine serum
SREBP: Sterol regulatory element-binding protein
WB: western blot
*wt*: Wild-type
