# Supplementary material for: Transcript, protein, metabolite and cellular studies in skin fibroblasts demonstrate variable pathogenic impacts of NPC1 mutations
Source: Orphanet J Rare Dis. 2020 Apr 5;15:85. doi: 10.1186/s13023-020-01360-5 (PMC7132889; doi:10.1186/s13023-020-01360-5)
Supplement: Supplementary file 1 — Additional file 1: Table S1. Cohort of NP-C1 patients. Correspondence with patients in Jahnova et al. [3] and full genotypes. Supplementary Figure 1: Scatter plot of NPC1 protein level semi-quantified byWestern blot analysis vs. object Pearson colocalization coefficient of NPC1 and LAMP2. Data labels indicate promoter haplotype allele combinations and deduced effects of the mutations in NPC1 protein. The dashed lines mark levels in controls (AVG ± SEM). Supplementary Figure 2: Mutant NPC1 residual function analyses. A scatter plot of Free Cholesterol / Cholesterol Ester ratio vs. direct filipin staining signal fold-change reflecting impaired cholesterol transport caused by NPC1 mutations. The combination of these markers suggest separation of severe infantile phenotypes. Solid circles represent individual patient cell lines and their grey level indicate clinical phenotype. Numbers in bold adjacent to the circles represent values of the LDL-cholesterol esterification rate assessed in the lab. of Dr. Vanier in Lyon. The dashed lines mark levels in controls (AVG ± SEM). [file 13023_2020_1360_MOESM1_ESM.pdf]

Table S1 – Cohort of NP-C1 patients. Correspondence with patients in Jahnova et al [3] and full genotypes.

Patient No.	Patient No. in Jahnova et al.	Clinical Phenotype	*NPC1* cDNA sequence change (ref. NM_000271.5)	Deduced NPC1 protein change (ref. NP_000262.2)	UC nmol/mg protein	CE nmol/mg protein
1	8. T2	Early infantile	c.[1812dupT];[3558delC]	p.[**A605Cfs*2**];[**A1187Rfs*55**]	202.4±2.3	35.0±3.0
2	-	Early infantile	c.[2746_2748delAAT];[3734_3735delCT]	p.[N916del];[**P1245Rfs*12**]	140.9±9.4	18.0±5.0
3	7. T2	Early infantile	c.[3182T>C];[3591+1G>A]	p.[I1061T];[T1176_S1196del;S1197_V1198ins15][$]	148.3±5.4	21.2±1.2
4	11. T2	Early infantile	c.[3557G>A];[3614C>A]	p.[R1186H];[T1205K]	330.3±7.2	21.3±0.9
5 Sb	10. T2	Late infantile	c.[826T>C];[3557G>A]	p.[Y276H];[R1186H]	196.7±15.3	19.7±4.4
6 Sb	10. T3	Late infantile	c.[826T>C];[3557G>A]	p.[Y276H];[R1186H]	195.6±7.8	25.5±1.8
7	1. T6	Late infantile	c.[3557G>A];[3557G>A]	p.[R1186H];[R1186H]	212.2±33.7	18.2±5.5
8	12. T3	Late infantile	c.[3557G>A];[3557G>A]	p.[R1186H];[R1186H]	141.5±0.4	20.2±3.8
9	9. T3	Late infantile	c.[3019C>G];[3557G>A]	p.[P1007A];[R1186H]	74.7±2.3	24.0±6.3
10	17. T4	Juvenile	c.[2861C>T];[3557G>A]	p.[S954L];[R1186H]	47.6±10.4	14.2±0.8
11	-	Juvenile	c.[2861C>T];[3557G>A]	p.[S954L];[R1186H]	60.4±10.4	16.6±1.2
12 Cs	20. T4	Juvenile	c.[2861C>T];[3557G>A]	p.[S954L];[R1186H]	78.4±2.7	43.2±21.7
13 Cs	12. T4	Juvenile	c.[2861C>T];[3557G>A]	p.[S954L];[R1186H]	64.0±8.2	15.0±0.7
14	9. T4	Juvenile	c.[1421C>T];[2072C>T]	p.[P474L];[P691L]	43.6±8.8	16.1±1.5
15	13. T4	Juvenile	c.[2849T>G];[3019C>G]	p.[V950G];[P1007A]	37.0±4.0	25.3±1.1
16	11. T4	Juvenile	c.[1232G>C];[3019C>G]	p.[R411P];[P1007A]	61.4±1.4	18.2±0.7
17	19. T4	Juvenile	c.[1232G>C];[2861 C>T]	p.[R411P];[S954L]	92.3±19.2	19.9±0.4
18	-	Juvenile	c.[527T>G];[2861C>T]	p.[L176R];[S954L]	66.6±4.2	19.0±1.9
19	14. T4	Juvenile	c.[352_353delAG];[3019C>G]	p.[**Q119Vfs*8**];[P1007A]	97.2±10.2	27.9±1.7
20	-	Juvenile	c.[808delG];[2861C>T]	p.[**V270Sfs*40**];[S954L]	55.3±12.5	13.0±0.3
21	-	Juvenile	c.[1723delG];[2861C>T]	p.[**E575Rfs*17**];[S954L]	89.2±0.8	28.6±2.3
22	15. T4	Juvenile	c.[1812dupT];[2861C>T]	p.[**A605Cfs*2**];[S954L]	52.6±1.6	13.4±0.8
23	3. T5	Adolescent/Adult	c.[2712delG];[2861C>T]	p.[**Q905Rfs*31**];[S954L]	53.8±11.8	20.2±0.3
24 Sb	18. T4	Adolescent/Adult	c.[2780C>T];[2780C>T]	p.[A927V];[A927V]	44.1±1.9	30.7±4.1
25 Sb	5. T5	Adolescent/Adult	c.[2780C>T];[2780C>T]	p.[A927V];[A927V]	47.8±4.5	23.0±0.1
26 Sb	6. T5	Adolescent/Adult	c.[2780C>T];[2780C>T]	p.[A927V];[A927V]	61.0±10.1	18.4±3.6
Control levels (n=3)		*wt*	*Wt*	*wt*	58,4 (39,6-77,6)	19,0 (10,9-25,1)

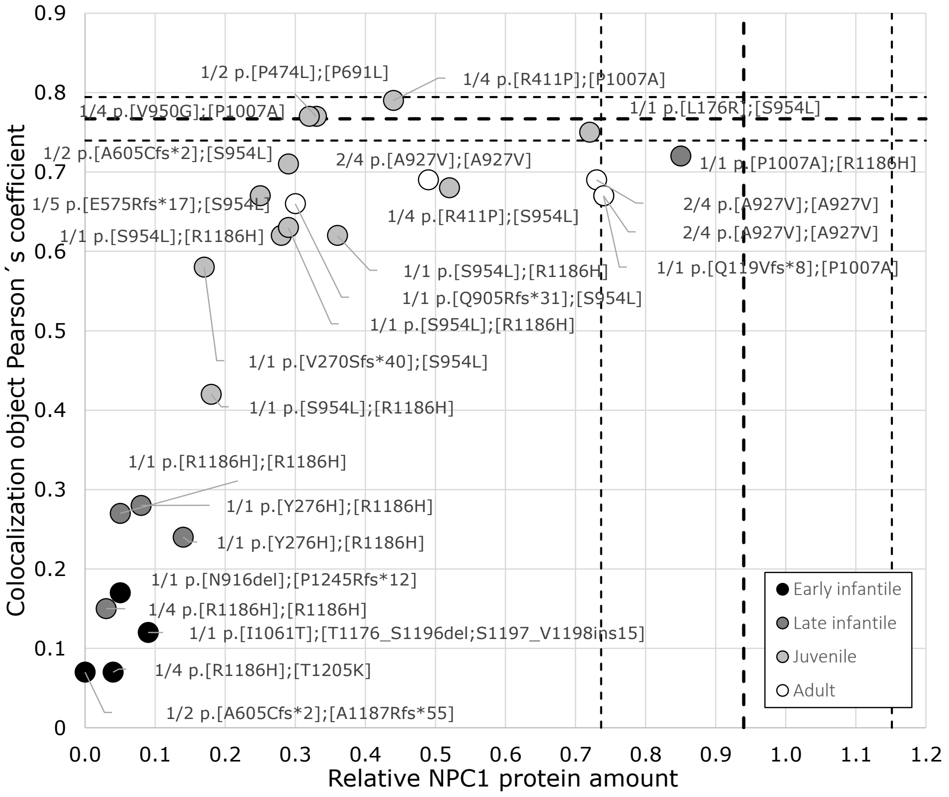

Supplementary Figure 1: Scatter plot of NPC1 protein level semi-quantified by Western blot analysis vs. object Pearson colocalization coefficient of NPC1 and LAMP2. Data labels indicate promoter haplotype allele combinations and deduced effects of the mutations in NPC1 protein. The dashed lines mark levels in controls (AVG ± SEM).

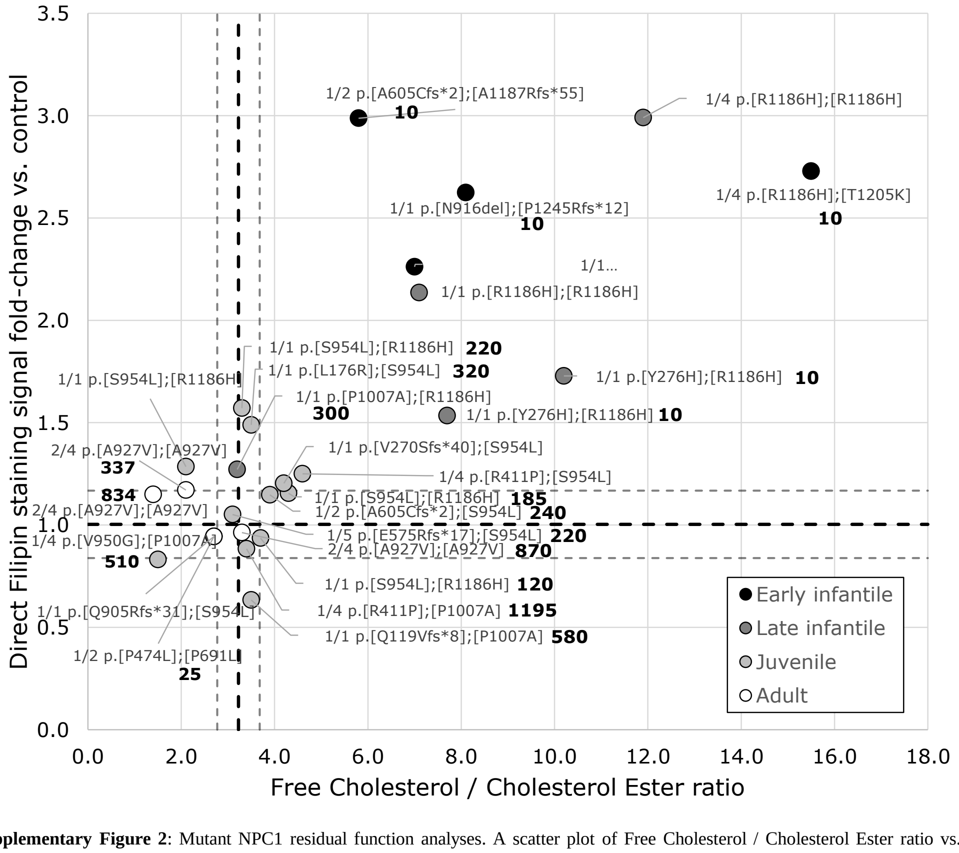

Supplementary Figure 2: Mutant NPC1 residual function analyses. A scatter plot of Free Cholesterol / Cholesterol Ester ratio vs. direct filipin staining signal fold-change reflecting impaired cholesterol transport caused by NPC1 mutations. The combination of these markers suggest separation of severe infantile phenotypes. Solid circles represent individual patient cell lines and their grey level indicate clinical phenotype. Numbers in bold adjacent to the circles represent values of the LDL-cholesterol esterification rate assessed in the lab. of Dr. Vanier in Lyon. The dashed lines mark levels in controls (AVG ± SEM).
